# Health Knowledge about Smoking, Role of Doctors, and Self-Perceived Health: A Cross-Sectional Study on Smokers’ Intentions to Quit

**DOI:** 10.3390/ijerph18073629

**Published:** 2021-03-31

**Authors:** Tianfeng He, Lefan Liu, Jing Huang, Guoxing Li, Xinbiao Guo

**Affiliations:** 1 Department of Occupational and Environmental Health Sciences, School of Public Health, Peking University, 38 Xueyuan Road, Beijing 100191, China; hetfnbcdc@163.com (T.H.); jing_huang@bjmu.edu.cn (J.H.); liguoxing@bjmu.edu.cn (G.L.); 2 Ningbo Municipal Center for Disease Control and Prevention, Ningbo 315010, China; 3 Center for Health Economics, School of Economics, University of Nottingham Ningbo China, Ningbo 315100, China; lefan.liu@nottingham.edu.cn

**Keywords:** health knowledge, smoking caused disease, secondhand smoke, cessation, intention to quit, low/middle income country

## Abstract

Limited empirical work has been done to compare the effects that health knowledge and advice from doctors have on smokers’ intentions to quit. This paper examines the association of smokers’ intentions to quit with health knowledge, advice from doctors, and self-perceived health. A sample of 2509 smokers aged 15–69 years old in Ningbo was used from China’s National Health Literacy Surveillance survey, conducted in 2018 and 2019. Respondents were asked whether they agree smoking causes stroke, heart attack, lung cancer; and heart diseases in adults, lung illnesses in children, and lung cancer in adults, by secondhand smoke, respectively. Using the logistic model, we found that knowing that smoking causes stroke and lung cancer more than doubles the odds of one’s intention to quit (OR = 2.705, *p* < 0.01), the effect of which is much greater than knowing that smoking causes lung cancer only (OR = 1.795, *p* < 0.01). Doctors’ advice to quit is more important than health knowledge, in terms of predicting smokers’ past cessation behaviours. In addition, smokers’ self-perceived health is negatively associated with their decisions to quit. This paper highlights that more resources should be directed towards training health care providers to advise smokers to quit, which might be more effective than health education alone.

## 1. Introduction

China has the largest population of smokers in the world, but has one of the lowest smoking cessation rates [1]. Data from the Global Adult Tobacco Survey (GATS), 2010, reveal that China was the second lowest among all GATS countries in terms of quit rates, measured by the proportion of former smokers among ever smokers [1]. The more recent GATS 2018 data in China showed that 16.1% of Chinese smokers planned or were thinking about quitting in the next 12 months, a figure unchanged from 2010 [2]. Behavioural theories, such as theory of planned behaviour [3], and transtheoretical model of behaviour change [4], consider behavioural intention as an important component in behaviour change. The primary goal of this study is to identify factors that could be targeted by policymakers to improve smokers’ intentions to quit.

A growing body of literature is looking at the factors that are associated with smokers’ intentions to quit. Previous studies have found that sociodemographic variables, including age, educational attainment, and family income, are associated with intentions to quit [5]. Smoking-related factors, such as knowledge about smoking-related diseases [6,7,8,9,10], home smoking rules [11], nicotine dependence, and previous quit attempts also predict smokers’ quit intentions. In addition, smoking rationalisation beliefs, also known as self-exempting beliefs, which justify or rationalise their smoking behaviours can also predict a lack of intention to quit [5]. Our paper is related to [12], a study conducted in 2010. Using a population-based sample from the International Tobacco Control (ITC) China Wave 1 survey, reference [12] examines the variations between current, former, and never smokers’ health knowledge about smoking. They also studied the impact of health knowledge on smokers’ intentions to quit. Recent work on this, however, is lacking. Our study aims to compare people’s perceptions and attitudes on health hazards of smoking over time by using more recent data sources, which include questions that follow the standard design in GATS. In addition, we extend our understanding on smokers’ intentions to quit by including two factors that were not considered in previous studies: advice from doctors and self-perceived health. We aim to identify the relative importance of health knowledge about smoking and doctors’ advice to quit smoking in motivating smokers to quit. This question is relevant to policymakers in allocating resources, as the former would require the resources be directed to educate smokers, but the latter suggests a more effective use of resources to train health care providers.

## 2. Materials and Methods

### 2.1. Study Site and Data Collection

Our data come from a sub-sample of the National Health Literacy Surveillance (NHLS) conducted in Ningbo, in 2018 and 2019 (see [13], for an overview on NHLS). Ningbo, located in the Yangtze River Delta in South China, was ranked the world’s fourth-largest port city in 2013 [14], and it is an important commercial and financial centre in South China. The population in Ningbo was 6.03 and 6.08 million in 2018 and 2019, respectively. In the two most recent surveys (2018 and 2019), a detailed questionnaire that is similar to GATS was incorporated, enabling us to examine the cessation behaviours of the smokers in Ningbo, China. This survey is representative of the permanent residents aged 15–69 years old who lived in Ningbo for more than 6 of the previous 12 months during the time of the survey. We used a stratified, multi-stage probability proportional to population size (PPS) sampling frame. For details, please refer to our earlier publication in [13]. We pooled the observations from the 2018 survey (covering 10 counties, 43 streets, 242 villages/communities involving 6581 individuals) and 2019 survey (covering 10 counties, 45 streets, 124 villages/communities involving 6340 individuals), and obtained a total sample of 12,921 respondents. All respondents who agreed to participate in the survey signed an informed consent form at the beginning of the survey. Patients or the public were not involved in the design, conduct, reporting, or dissemination plans of our research. Our final sample included 12,915 respondents (aged 15–69) surveyed in 2018 and 2019, of whom we have complete information on the key variables of interest. 

### 2.2. Measures

#### 2.2.1. Smoking Status and Cessation Classification

Respondents who were smoking at the time of the survey were classified as current smokers. Those who smoked before but were not smoking at the time of the survey, were classified as former smokers. Those who never smoked were classified as never smokers.

For current smokers, they were asked whether they were planning to quit in the next month, 12 months, beyond 12 months, or not at all, and the responses were categorized into two groups: 1 “To quit at any point” and 0 “do not want to quit”. We treated a response of “I do not know” as missing. Other than intention to quit, the survey also asked whether the smoker attempted to quit in the past 12 months, allowing us to construct the past cessation behaviours among smokers who ever smoked. Respondents were also asked whether they were advised to quit by health care providers in the past 12 months when seeing a doctor, and the responses were categorized into two groups: 1 “Doctors’ advice to quit” and 0 “no/not applicable”.

#### 2.2.2. Health Knowledge about Smoking

Respondents were asked whether they agreed (to their knowledge) that smoking causes stroke, heart attack, lung cancer in smokers; and whether secondhand smoke causes heart diseases, lung cancer in children, and lung cancer in adults. Responses were coded as 0 “no/do not know” versus 1 “yes”. A health knowledge scale was also created by summing the number of “yes” responses across the 6 diseases (ranges 0 to 6). In addition, a respondent was defined as having adequate health knowledge about smoking if the respondent agreed that smoking caused the three diseases (stroke, heart attack, and lung cancer).

#### 2.2.3. Demographic Variables and Health Conditions

Demographic and socioeconomics variables used as covariates included region of residence (rural/urban), gender, household annual income per capita (2019 price, Consumer Price Index adjusted), number of household members, occupation (public sectors; farmers; manual labourers; and other), age (15–44; 45–59; 60–69), and education level (illiterate; elementary/middle school; high school; and college or above). 

Respondents were asked whether they had any chronic disease and the type of the disease, if any, including hypertension, heart problems, cerebrovascular diseases, diabetes, malignant tumour (cancer), and other diseases if not listed. In addition, respondents were asked to rate their general health as being: (1) excellent, (2) good, (3) fair, (4) poor, or (5) very poor, which we further combined into two groups: 1 “self-reported good health” if the response was good/excellent, and 0 otherwise.

### 2.3. Statistical Analyses

A smoker’s intention to quit was used as the primary dependent variable. We also used other variables that characterized smokers’ cessation behaviours in different stages, including whether they intended to quit in the next 12 months, whether they attempted to quit in the past 12 months, and whether they quit smoking in the past 12 months. In so doing, we have a comprehensive picture of a smoker’s decision to quit at different stages. The key explanatory variables of interest included health knowledge about smoking, whether they were advised to quit by doctors, and self-reported health status. A series of controls included demographic and socio-economic status factors mentioned earlier. Logistic regression was used for our binary dependent variable and odds ratios (OR) were reported. All statistical analyses were performed using StataCorp LLC’s Stata Statistics Software version 15.0 [15].

## 3. Results

### 3.1. Health Knowledge about Smoking and Secondhand Smoke

First, we examined the level of knowledge that respondents had about the health hazards caused by smoking or by secondhand smoke; the results are presented in Table 1. As seen in the last column, overall, 90% of our respondents agreed that smoking causes lung cancer, but only 54% (or 51%) of respondents agreed that smoking causes stroke (or heart attack). The pattern is similar regarding the health hazards caused by secondhand smoke. The percentage of respondents who agreed that secondhand smoke causes lung cancer in adults is 88%, which dropped to 77% responding to the question that secondhand smoke causes lung illnesses in children. Only 54% agreed that secondhand smoke causes heart diseases in adults.

Next, we examined the variations in health knowledge among current, former, and never smokers (columns 1–3). Overall, current smokers agree with fewer disease types caused by smoking (mean = 3.95 out of 6) compared to former smokers (mean = 4.10; *p* = 0.028) and never smokers (mean = 4.19; *p* < 0.010) and the same is true for each individual disease type. The proportion of respondents who agreed that secondhand smoke causes heart disease was about 53% and did not differ statistically by smoking status of our respondents.

### 3.2. Sample Characteristics of Smokers

We present the demographic and socioeconomic characteristics of smokers and compare with the other two groups in Table 2. Current smokers are mainly males (95%). An average smoker is 51 years old and lives in a household with three members. The annual household income is, on average, 36,946 yuan per capita after adjusted for household size. This figure is higher than the 2019 national average of 30,733 yuan [16]. As mentioned earlier, Ningbo city is a port city, as well as a commercial and financial centre in South China. Thus, on average our respondents have better socioeconomic status than the national average. The 2018 GDP per capita in Zhejiang province, in which Ningbo belongs to, ranks fifth among 31 provinces in China [17]. Compared to people who never smoke, current smokers are more likely to reside in rural areas, are older, poorer, less educated, and more likely to be farmers and manual labourers. Compared to former smokers, current smokers are younger, wealthier, better educated, and more likely to be manual labourers.

Contrasts arise when we compare the health status between current smokers and never/former smokers. Overall, the never smokers are the healthiest. Although the prevalence rate for having any chronic disease is slightly higher among current smokers than among never smokers (28% vs. 25%), the two groups have similar prevalence rates for hypertension, heart diseases, cerebrovascular diseases, and cancer. The former smokers have the worst health profile. About 56% of former smokers self-reported good health (11 percentage points lower than the other two groups) and 42% self-reported having at least one chronic disease (i.e., 58% have no chronic diseases). There are two possibilities in explaining why current smokers have better health profiles than former smokers. Current smokers may have not yet developed any diseases, thus self-reporting good health. Alternatively, current smokers have developed diseases, but they are in denial about their worse health status. In other words, it is the disease diagnosis or perception of health that is associated with a smoker’s quitting behaviour. We will examine the role that self-perceived health plays later.

Indeed, among 2806 current smokers, only 38% responded that they intended to quit at any point, among which, less than half-planned to quit next month or in 12 months. Regarding past cessation behaviours, about 29% of smokers attempted to quit in the past 12 months, and about half of the smokers reported being advised to quit by a health care provider when seeing a doctor. This figure, however, reaches two-thirds among former smokers. The proportion of current smokers with adequate health knowledge about smoking is 42%. This proportion is slightly higher among former and never smokers.

### 3.3. Baseline Results on the Effect of Health Knowledge on Smoking and Intention to Quit

The logistic regression on intention to quit is presented in Table 3. The first equation in column (1) reveals that having adequate health knowledge about smoking is associated with an increase in the odds of intention to quit. On average, those who agree that smoking causes stroke, heart diseases, and lung cancer have higher odds of intending to quit in the future by 68%, relative to those who were not fully aware of the health hazards of smoking. According to column (2), doctors’ advice to quit is associated with higher odds of intention to quit by 50%. In contrast, those smokers who self-reported good health are less likely to consider quitting as revealed in column (3). In column (4), which is our baseline specification, we include the demographic and socioeconomic covariates and the coefficients of our key variables of interest are similar. The full results for column (4) are reported in column (1) in Table A1 in Appendix A. All covariates have expected signs. An older smoker on average has a lower willingness to quit, and those with an education level of college or above are significantly more likely to consider quitting in the future than the illiterate. Relative to the public sector workers, smokers who are farmers, manual labourers, or other are significantly less likely to consider quitting.

### 3.4. Heterogeneous Results

We also examine the extent to which the effects of health knowledge, doctors’ advice to quit, and self-reported health status vary by the region, gender, and age of the smoker. To this end, we run the estimation separately for the rural/urban, male/female, young/old subsamples, and the results are presented in Table 4. The “young” group includes those aged 16–54 and the “old” group includes those aged 55–69, which roughly divides the full sample into equal sizes. 

In terms of health knowledge effect, it seems to be more pronounced in rural than in urban areas. The effect of doctors’ advice to quit is similar in our rural and urban subsamples. The negative effect of self-reported good health is pronounced in both urban and rural subsamples. We only have 47 female smokers, and few variables are significant (with large standard errors). We do not report these results, but they are available upon request. The results for the sample of males are almost identical to what we have in Table 3. In columns (5)–(6) in Table 4, we examine the results by age. The health knowledge effect is stronger among the older smokers, among whom the effect of doctors’ advice is also greater. In addition, although self-reported health is negatively associated with a smoker’s intention to quit, it is mainly driven by old smokers (i.e., aged 55–69). 

We also look at the effects of covariates, which are reported in columns (2)–(6) in Table A1 in Appendix A. In the rural subsample, there is a significant year effect where smokers surveyed in 2019 are significantly more likely to consider quitting in the future. We think this might be relevant to the local government’s policy in banning smoking in public areas, which started in 2018, and was strengthened in 2019. Indeed, when we check the proportion of respondents who reported noticing smoking in public areas, there is a greater decline from 2018 to 2019 in rural areas than in urban areas (see Table A3 in Appendix A). The policy might have triggered more rural smokers’ intentions to quit in 2019 relative to 2018.

### 3.5. The Role of Health Knowledge about Smoking

In Table 5, we study the effect that the type and level of health knowledge has on a smoker’s intention to quit. In column (1), we replace the binary health knowledge variable with an alternative that is coded one, only if the respondents agree with the aforementioned six statements. In column (2), we replace the binary variable with six dummies, each indicating the agreement with a disease type. Only “smoking causes stroke” and “smoking causes lung cancer” are significant, suggesting that smokers who agree smoking causes stroke or lung cancer increases the odds of intention to quit and the effect is similar to the estimates in column (1). In column (3), we include the continuous health knowledge scale: a “one” unit increase in the knowledge scale is associated with an increase in the odds by 16%. Lastly, in column (4), we replace the scale variable with two dummy variables, indicating exclusively smokers’ knowledge being that “smoking causes lung cancer, but not stroke” and “smoking causes lung cancer and stroke”. It is rare that a smoker agreed that smoking causes stroke, but not lung cancer. We dropped 36 of such observations from the sample. Compared to the reference group (who do not agree smoking causes lung cancer or stroke), knowing smoking causes lung cancer but not stroke increases the odds of intention to quit by 80%; while knowing smoking causes lung cancer as well as stroke raises the odds of intention to quit by 171%.

### 3.6. The Role of Doctors and Self-Perceived Health

In this subsection, we explore the role of doctors and self-perceived health in relation to the cessation behaviour of smokers. We consider three alternative variables as the outcome variable: (1) whether a current smoker intended to quit in the next 12 months. (2) Whether an ever smoker (i.e. current smokers or former smokers) attempted to quit in the past 12 months (excluding former smokers who have been abstinent for more than 12 months). (3) Whether an ever smoker quitted in the past 12 months (excluding former smokers who have been abstinent for more than 12 months). Thus, we contrast the smokers’ intentions to quit with past cessation behaviour in the time window of the same length (12 months). The results are presented in Table 6. 

Similar to our baseline results, health knowledge about smoking is positively associated with a current smoker’s intention to quit in the next 12 months (Table 6, column (1)). However, it has little effect on whether an ever smoker attempted to quit or actually quitted in the past 12 months (Table 6, columns (2)–(3)). The estimates for doctors’ advice to quit, however, are similar across the three regressions. Similarly, a smoker’s past cessation behaviour is significantly associated with the smoker’s health status (measured by self-reported health status). 

## 4. Discussion

Our study shows among 2509 current smokers (aged 15–69) surveyed in Ningbo, a city located in the Yangtze River Delta in South China; the proportion who intended to quit at any point in the future was about 38% in 2018–2019. We examined the association of intention to quit with a smoker’s health knowledge about smoking, doctors’ advice to quit, and self-perceived health, controlling for demographic and socioeconomic factors.

**Figure A1:**
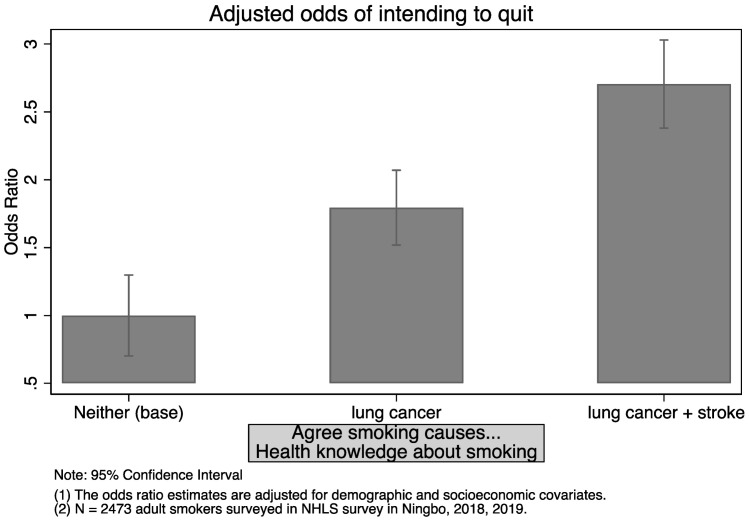
Adjusted odds of intending to quit.

We observed a significantly lower level of health knowledge about smoking among current smokers compared to the former smokers and those who never smoked. This finding is consistent with [12], using the Wave 1 (2006) ITC China survey with a sample of 1000 adults in six cities in China. Similar to the GATS 2018 China results [2], we found that the majority (90%) of adults are aware that smoking causes lung cancer, but fewer (51–54%) are aware that smoking also causes stroke or heart diseases. These figures represent significant improvement compared to that in 2010. In the GATS 2010 China results, about 78% of adults agreed that smoking caused lung cancer and only 27% (39%) agreed that smoking caused stroke (heart attack) [1]. Levels of health knowledge among Chinese smokers, notwithstanding, were considerably lower than levels previously reported in western countries. For example, in Canada, which has some of the most progressive tobacco control policies in the world, approximately 83% of smokers agreed that smoking causes stroke and 91% agreed that smoking causes heart disease, in 2002 [18]. The corresponding figures in our sample of smokers are 52% and 47%. We examined the extent to which this knowledge gap has implications on smokers’ intentions to quit. We found that the effect of knowing smoking causes lung cancer and stroke, relative to no such knowledge, potentially doubles the odds of intending to quit, outweighing the effect of knowing smoking causes lung cancer only. We contrast this finding by plotting the associated odds ratio in Figure A1 in Appendix B.

**Figure A2:**
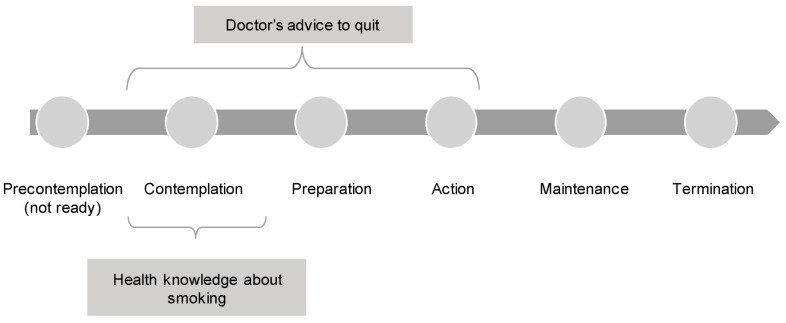
A schematic showing how health knowledge and doctors’ advice to quit influence different stages of quitting based on transtheoretical model of behaviour change.

We extended previous studies by examining the relative importance of health knowledge and doctors’ advice in affecting the cessation behaviour of smokers in Table 6. We found that health knowledge is associated with a smoker’s intention to quit, but is not associated with their past attempts of quitting or whether a smoker quit in the time window of 12 months. In contrast, not only is doctors’ advice associated with a current smoker’s intention to quit, but it is also associated with a smoker’s action to quit. Our findings are consistent with the transtheoretical model of health behaviour change. According to [18], to progress from pre-contemplation (i.e., do not want to quit) to contemplation (i.e., intended to quit), the pros of changing must increase. To progress from contemplation to action, the cons of changing must decrease. Health knowledge potentially motivates a smoker to transition from precontemplation stage to contemplation stage, but plays a limited role in motivating smokers into action. In contrast, doctors’ advice to quit might play a more important role in helping the smokers to progress to the action stage. We illustrate in a schematic (Figure A2 in Appendix B) how health knowledge and advice from doctors potentially influence a smoker’s decision to quit at different stages. This is also evidenced in a recent study that finds cessation advice from health care professionals helps those in the contemplation and preparation stage [19]. 

The important role of doctors’ advice to quit might be an outcome of better knowledge about the health risks of smoking. To address this concern, we test whether the effect of doctors’ advice depends on smoker’s health knowledge, by including interaction terms. The interaction term is not significantly different from one (these results are not reported by available upon request). In other words, doctors’ advice to quit is associated with a smoker’s intention to quit regardless of their health knowledge about smoking. 

We also observe a relationship between self-reported health status and smokers’ intentions to quit. Those who self-reported good health are less likely to consider quitting. This might arise for two reasons. Firstly, smokers’ belief about the health risks of smoking influences their decision to quit. This is in line with [12,20,21], where smokers who reported greater worry about the future health effects of smoking and smokers who reported health benefits from quitting are likely to consider quitting. On the other hand, self-reported health status is a good measure of the general health status of an adult. The second reason, therefore, is that worse physical health motivates smokers to quit. To test the extent to which the two explanations hold, we add number of chronic diseases in our model to control for the physical health of smokers as an objective health indicator. These results are presented in Panel A, Table A2 in the Appendix A. 

Self-reported health status remains significant in columns (1)–(2) in Table A2 in the Appendix A, indicating smokers are indeed influenced by their beliefs (about their own health), and this effect cannot be explained by their more objective health statuses. In column (3) in Table A2 in the Appendix A, when the outcome of quitting instead of intention or attempt is considered, self-reported good health is no longer significant but the estimate on number of chronic diseases is highly significant. This is reasonable given “to have quit” for a period of time would often require more external assistance than beliefs alone. To identify which disease types are more likely to “trigger” a smoker to quit, we replaced the number of chronic diseases with five dummy variables, indicating specific types of diseases (see Panel B in Table A2 in the Appendix A). The likelihood of quitting increased when smokers were diagnosed with cerebrovascular diseases or cancer. For example, it shows if a smoker was diagnosed with cerebrovascular disease, the odds of quitting in the past 12 months increase by 277%.

The above findings highlight the importance of disease prevention and control. Smoking doubles the risk of having a heart attack and it causes 84% of deaths from lung cancer and 83% of deaths from chronic obstructive pulmonary disease (COPD) [22]. This knowledge was more relevant when a smoker had the diagnosis with a disease instead of being informed of the risk of developing the illness. Thus, the quitting attempts are more likely to be triggered by health care providers than by a smoker’s own knowledge about the harm of smoking. Our view is in line with “although awareness and acceptance of the health risks of smoking may not be a sufficient condition for quitting, it is likely a necessary one for most smokers and serves an important source of motivation” [23]. 

It is worth nothing that our finding does not imply medical doctors hold a central role in motivating smokers to quit. In countries where quitting programs have been successful, substantial socio-environmental changes were made along with tobacco control policies and televised antismoking advertising [24,25]. Our finding suggests advice from health care providers is likely to supplement these campaigns. A similar study to ours, using data of Korean adult smokers, found that a doctors’ advice to quit alone increases the likelihood of a smoker’s intention to quit within 1 month (OR = 2.14, *p* < 0.05), but not within 6 months or someday in the future. However, it is likely to increase the effect of a significant other’s advice [26]. In other words, smokers who had been advised to quit smoking by both significant others and medical professionals are more likely to consider quitting than those who had been advised to quit by significant others only. 

There are several limitations in our study, and our findings should be interpreted with caution. Firstly, this is a cross-sectional study. There might exist factors that influence a smoker’s intention to quit and the likelihood of getting advice from doctors to quit smoking. It is likely that people living in communities with better access to health facilities are more likely to be advised by doctors to quit smoking. They are also more likely to consider quitting in the future, because there is a higher level of trust among residents in the same communities. Previous studies found that being a smoker was associated with a higher level of perceived income inequality, lower perception of relative material well-being, and living in a community with a lower degree of trust and safety [27]. Information of community characteristics of smokers’ residence is likely to correct this problem, and we consider it a direction for future research. Secondly, our data are not representative nationally, and Ningbo represents one of the cities with better economic development in China. We do not think our findings apply to all regions in China. Lastly, in order to demonstrate that smokers are triggered to quit based on misbelieves of their health statuses, we included chronic diseases as objective measures of health. They can still be argued to be subjective because some illnesses might not be diagnosed.

## 5. Conclusions

We examined smokers’ knowledge about the health hazards of smoking in China, in 2018–2019, by using NHLS data from Ningbo, a city located in the Yangtze River Delta in South China. Although the majority of the general public are aware that smoking causes lung cancer, only half of them agree that smoking causes heart attack or stroke. This knowledge gap is strongly associated with smokers’ intentions to quit. However, when we examined smokers’ actions in quitting, using variables indicating their past cessation behaviours, the importance of health knowledge about smoking diminishes. Smokers’ past cessation behaviours are more related to whether smokers were advised to quit by health care providers. Self-perceived health also has a role to play. It appears that people quit smoking when they perceive their health is getting worse. The policy implications of our study are two-fold. Firstly, it is of public health importance to encourage health professionals to advise smokers to quit with effective assistance. For example, in the UK, health care professionals are required to identify and refer smokers using the method known as Very Brief Advice (VBA) involving “(1) ASK and record smoking status; is the patient a smoker, ex-smoker or non-smoker? (2) ADVISE on the best way of quitting; the best way of stopping smoking is with a combination of stop smoking aids and specialist support; (3) ACT on patient response; build confidence, give information, refer, and prescribe” [22]. Similar procedures can be adopted in China. Secondly, in light of the disparity in socioeconomic groups, in terms of smoking prevalence and intention to quit, when designing policies, certain groups should be targeted. For example, rural smokers should be targeted with more intensive health knowledge interventions, given they are more responsive than their urban counterparts, concerning the effect of health knowledge. 

## Figures and Tables

**Table 1:** Health knowledge about smoking, by smoking status (% (N)).

Health Knowledge about Smoking	(1)	(2)	(3)	(4)
	Never Smokers	Former Smokers	Current Smokers	Overall
Adults who agree smoking causes:				
Lung cancer (%)	91.5	89.2	86.3	90.2 ^a,b^
	(9136)	(973)	(2806)	(12915)
Stroke (%)	55.4	53.9	51.6	54.4 ^a^
	(9136)	(973)	(2806)	(12915)
Lung cancer (if … agrees smoking causes stroke) (%)	97.9	96.9	97.2	97.7
	(5059)	(524)	(1449)	(7032)
Heart attack (%)	51.8	51.5	47.2	50.8 ^a,b^
	(9136)	(973)	(2806)	(12915)
Adults who agree secondhand smoke causes:				
Lung cancer in adults (%)	89.0	85.8	83.6	87.6 ^a^
	(9136)	(973)	(2806)	(12915)
Heart diseases in adults (%)	53.8	53.6	52.3	53.5
	(9136)	(973)	(2806)	(12915)
Lung illnesses in children (%)	78.1	76.8	74.2	77.2 ^a^
	(9136)	(973)	(2806)	(12915)
Number of items agreed (0-6)	4.192	4.090	3.954	4.133 ^a,b^
	(9136)	(973)	(2806)	(12915)

Notes: (1) numbers in parentheses indicate the number of observations in each entry. (2) ^a^ indicates the difference between current and never smokers is significant at *p* < 0.05; ^b^ indicates the difference between current and former smokers is significant at *p* < 0.05.

**Table 2:** Sample mean by smoking status of National Health Literacy Surveillance (NHLS) survey in Ningbo.

Variables	(1)	(2)	(3)	(4)
	Never Smokers	Former Smokers	Current Smokers	Total
	Mean	Mean	Mean	Mean
Demographics				
Surveyed in 2019 (=1)	0.490	0.519	0.484	0.491
Urban (=1)	0.617	0.528	0.545	0.594
Male (=1)	0.287	0.940	0.975	0.486
Age in years	48.202	54.715	51.589	49.429
1:Aged 15–44	0.372	0.181	0.247	0.331
2:Aged 45–59	0.373	0.392	0.472	0.396
3:Aged 60–69	0.255	0.428	0.281	0.273
Household size	2.857	2.856	2.847	2.855
Annual household income per capita (1000 Yuan)	39.508	33.058	36.947	38.467
Education level				
Illiterate	0.087	0.068	0.049	0.077
Elementary/middle	0.511	0.671	0.636	0.550
High school	0.162	0.157	0.191	0.168
College or above	0.239	0.104	0.125	0.204
Job status				
Public sectors	0.133	0.090	0.078	0.118
Farmers	0.239	0.357	0.298	0.261
Manual labourers	0.167	0.193	0.242	0.186
Other	0.461	0.360	0.382	0.436
Health status				
Self-reported good health (=1)	0.673	0.560	0.672	0.665
Any chronic disease (=1)	0.254	0.425	0.277	0.272
Has hypertension (=1)	0.180	0.278	0.189	0.190
Has heart diseases (=1)	0.013	0.032	0.013	0.015
Has cerebrovascular disease (=1)	0.007	0.031	0.010	0.010
Has cancer (=1)	0.009	0.023	0.003	0.009
Has other diseases (=1)	0.027	0.041	0.031	0.029
Smoking related				
Smoke daily (=1)	/	/	0.891	0.891
Intended to quit (=1)	/	/	0.383	0.383
1:to quit in 1 m	/	/	0.053	0.053
2:to quit in 12 m	/	/	0.099	0.099
3:to quit in 12–24 m	/	/	0.194	0.194
4:do not want to quit	/	/	0.654	0.654
Adequate health knowledge (=1)	0.452	0.444	0.416	0.444
Doctors’ advice to quit (=1)	/	0.664	0.501	0.516
Attempted to quit past 12 m (=1)	/	/	0.288	0.288
Observations	N = 9136	N = 973	N = 2806	N = 12,915

Notes: (1) the response to the question on intention to quit is grouped into “to quit in 1 month”, “to quit in 12 months”, “to quit in 12–24 months” and “not at all”. (2) Adequate health knowledge is coded 1 if a respondent agrees smoking causes stroke, heart attack and lung cancer, 0 otherwise. (3) Doctors’ advice to quit is coded 1 if an ever smoker was advised to quit in the past 12 months, 0 otherwise.

**Table 3:** Logistic regression on intention to quit: baseline results.

Variables	(1)	(2)	(3)	(4)
	OR	OR	OR	OR
Adequate health knowledge	1.683 ***	1.626 ***	1.645 ***	1.460 ***
	(0.140)	(0.137)	(0.139)	(0.130)
Doctors’ advice to quit		1.501 ***	1.466 ***	1.578 ***
		(0.126)	(0.124)	(0.139)
Self-reported good health			0.830 **	0.756 ***
			(0.075)	(0.071)
Covariates adjusted	No	No	No	Yes
R-squared	0.012	0.019	0.020	0.057
N	2509	2509	2509	2509

Notes: (1) dependent variable is binary, =1 if a smoker intends to quit at any point, = 0 if does not intend to quit. The sample mean of the dependent variable is 0.383. (2) Covariates include survey year, gender, age, household size, annual household income per capita (log), education, job status, and county dummies. See column 1 in Table A1 in the Appendix A for full reports. (3) Exponentiated coefficients (odds ratios) are reported; standard errors in parentheses. ** *p* < 0.05, *** *p* < 0.01.

**Table 4:** Logistic regression on intention to quit: heterogeneity results.

Variables	(1)	(2)	(3)	(4)	(5)	(6)
	Rural	Urban	Males	Females	Young	Old
Adequate health knowledge	1.766 ***	1.234 *	1.443 ***	/	1.325 **	1.645 ***
	(0.242)	(0.147)	(0.130)	/	(0.158)	(0.228)
Doctors’ advice to quit	1.698 ***	1.522 ***	1.557 ***	/	1.404 ***	1.957 ***
	(0.228)	(0.184)	(0.139)	/	(0.165)	(0.277)
Self-reported good health	0.770 *	0.754 **	0.766 ***	/	0.828	0.686 ***
	(0.107)	(0.098)	(0.073)	/	(0.107)	(0.096)
Covariates adjusted	Yes	Yes	Yes	Yes	Yes	Yes
Dependent variable mean	0.367	0.389	0.376	0.489	0.423	0.325
R-squared	0.072	0.062	0.056	0.528	0.064	0.059
Obs	1148	1361	2458	47	1375	1134

Notes: (1) dependent variable is binary, =1 if a smoker intends to quit at any point, =0 if does not intend to quit. (2) Covariates include survey year, gender, age, household size, annual household income per capita (log), education, job status, and county dummies. See Table A1 in the Appendix A for full reports. (3) In column (4), we do not report the results because the sample size is too small (N = 47), and most coefficients are insignificant and have large standard errors. (4) Young indicates “aged 16–54” and Old indicates “aged 55–69”. (5) Exponentiated coefficients (odds ratios) are reported; standard errors in parentheses * *p* < 0.10, ** *p* < 0.05, *** *p* < 0.01.

**Table 5:** Logistic regression on intention to quit: health knowledge effects.

Variables	(1)	(2)	(3)	(4)
	OR	OR	OR	OR
Doctors’ advice to quit	1.592 ***	1.548 ***	1.552 ***	1.572 ***
	(0.141)	(0.138)	(0.138)	(0.142)
Self-reported good health	0.761 ***	0.769 ***	0.765 ***	0.777 ***
	(0.071)	(0.072)	(0.072)	(0.074)
Adequate knowledge	1.482 ***			
	(0.134)			
Agree smoking causes				
… stroke		1.547 ***		
		(0.198)		
… heart disease		0.980		
		(0.132)		
… lung disease		1.491 **		
		(0.278)		
Agree secondhand smoke causes				
… heart diseases in adults		1.006		
		(0.129)		
… lung illnesses in children		1.004		
		(0.135)		
… lung cancer in adults		1.173		
		(0.212)		
Number of items (0-6)			1.156 ***	
			(0.027)	
Knowledge gap (No knowledge)				ref.
Knowledge … lung cancer only				1.795 ***
				(0.299)
Knowledge … lung cancer and stroke				2.705 ***
				(0.437)
Covariates adjusted	Yes	Yes	Yes	Yes
R-squared	0.058	0.066	0.063	0.069
N	2509	2509	2509	2473

Notes: (1) dependent variable is binary, =1 if a smoker intends to quit at any point, =0 if does not intend to quit. The sample mean of the dependent variable is 0.383. (2) Adequate knowledge =1 if a respondent agrees the health effects of smoking in all 6 items, 0 otherwise. (3) The same covariates as in Table 3 are included: survey year, gender, age, household size, annual household income per capita (log), education, job status, and county dummies. (4) The sample size is smaller in column 4 because we drop 36 observations who agree smoking causes stroke but not cancer. (5) Exponentiated coefficients (odds ratios) are reported; standard errors in parentheses. ** *p* < 0.05, *** *p* < 0.01.

**Table 6:** Logistic regression on varying cessation behaviours.

Variables	(1)	(2)	(3)
	Intend to Quit 1y	Attempted 1y	Quitted 1y
	OR	OR	OR
Adequate health knowledge	1.392 ***	1.118	0.905
	(0.159)	(0.092)	(0.124)
Doctors’ advice to quit	1.876 ***	2.094 ***	1.903 ***
	(0.218)	(0.172)	(0.264)
Self-reported good health	0.713 ***	0.737 ***	0.748 **
	(0.083)	(0.062)	(0.102)
Covariates adjusted	Yes	Yes	Yes
Dependent variable mean	0.165	0.349	0.089
R-squared	0.043	0.055	0.051
N	2506	3052	3052

Notes: (1) dependent variable in column 1 =1 if intend to quit in the next 12 months; dependent variable in column 2 =1 if attempted to quit in the past 12 months (former smokers included); dependent variable in column 3 =1 if quitted smoking in the past 12 months (former smokers included). (2) Covariates include survey year, gender, age, household size, annual household income per capita (log), education, job status and county dummies. See Table A4 in the Appendix A for full reports. (3) Exponentiated coefficients (odds ratios) are reported; standard errors in parentheses. ** *p* < 0.05, *** *p* < 0.01.

## Data Availability

The data used and/or analysed in the current study are not publicly available because restrictions apply to the availability of the data. Data are, however, available from the corresponding author by reasonable requests, and with permission from the Ningbo Municipal CDC.

## Appendix A

**Table A1:** Logit regression on intention to quit: Full results for the full and different subsamples.

Variables	(1)	(2)	(3)	(4)	(5)	(6)
Samples	All	Rural	Urban	Males	Young	Old
Adequate health knowledge	1.460 ***	1.766 ***	1.234 *	1.443 ***	1.325 **	1.645 ***
	(0.130)	(0.242)	(0.147)	(0.130)	(0.158)	(0.228)
Doctors’ advice to quit	1.578 ***	1.698 ***	1.522 ***	1.557***	1.404 ***	1.957 ***
	(0.139)	(0.228)	(0.184)	(0.139)	(0.165)	(0.277)
Self-reported good health	0.756 ***	0.770 *	0.754**	0.766 ***	0.828	0.686 ***
	(0.071)	(0.107)	(0.098)	(0.073)	(0.107)	(0.096)
2019 (=1) vs. 2018 (=0)	1.006	1.406 **	0.838	1.017	0.989	1.033
	(0.087)	(0.202)	(0.108)	(0.089)	(0.115)	(0.141)
Urban	1.034			1.021	1.000	1.030
	(0.103)			(0.102)	(0.133)	(0.157)
Male	0.546 **	0.745	0.451 **		0.633	0.467 *
	(0.163)	(0.380)	(0.173)		(0.279)	(0.194)
Age/10	0.798 ***	0.806 ***	0.793 ***	0.802 ***	0.778 ***	0.844
	(0.038)	(0.059)	(0.049)	(0.038)	(0.063)	(0.141)
Household size	1.016	1.019	0.996	1.019	1.020	0.991
	(0.037)	(0.054)	(0.052)	(0.037)	(0.053)	(0.054)
Household annual income pc (log)	1.004	1.028	0.964	1.002	0.967	1.085
	(0.029)	(0.043)	(0.040)	(0.029)	(0.034)	(0.064)
Education level: illiterate	ref.	ref.	ref.	ref.	ref.	ref.
Elementary/middle	1.318	1.667 *	0.837	1.350	2.009	1.331
	(0.279)	(0.477)	(0.300)	(0.304)	(1.336)	(0.326)
High school	1.562 *	1.636	1.184	1.617 *	2.576	1.305
	(0.373)	(0.555)	(0.452)	(0.407)	(1.745)	(0.404)
College or above	1.602 *	1.541	1.215	1.704 *	2.826	0.619
	(0.428)	(0.646)	(0.492)	(0.474)	(1.942)	(0.298)
Job status: public sectors	ref.	ref.	ref.	ref.	ref.	ref.
Farmers	0.637 **	0.803	0.513 ***	0.649 **	0.437 ***	0.968
	(0.120)	(0.242)	(0.131)	(0.124)	(0.106)	(0.373)
Manual labourers	0.712 *	0.946	0.533 ***	0.747	0.563 ***	1.072
	(0.132)	(0.285)	(0.130)	(0.139)	(0.124)	(0.421)
Other	0.789	1.018	0.663 *	0.815	0.697 *	0.960
	(0.134)	(0.296)	(0.144)	(0.140)	(0.139)	(0.364)
Covariates adjusted	Yes	Yes	Yes	Yes	Yes	Yes
Dependent variable mean	0.383	0.367	0.389	0.376	0.423	0.325
R-squared	0.057	0.072	0.062	0.056	0.064	0.059
Obs	2509	1148	1361	2458	1375	1134

Notes: (1) dependent variable is binary, =1 if a smoker intends to quit at any point, =0 if does not intend to quit. The sample mean of the dependent variable is 0.383. (2) Constant is not reported. (3) Young indicates “aged 16-54” and Old indicates “aged 55–69”. (4) Exponentiated coefficients (odds ratios) are reported; standard errors in parentheses * *p* < 0.10, ** *p* < 0.05, *** *p* < 0.01.

**Table A2:** Logit estimation on varying cessation behaviours.

Variables	(1)	(2)	(3)
	Intend to Quit 1y	Attempted 1y	Quitted 1y
Panel A:			
Adequate health knowledge	1.388 ***	1.121	0.902
	(0.158)	(0.092)	(0.124)
Doctors’ advice to quit	1.876***	2.054 ***	1.806 ***
	(0.219)	(0.170)	(0.253)
Self-reported good health	0.713 ***	0.780 ***	0.842
	(0.086)	(0.069)	(0.120)
No. of chronic diseases (0-5)	1.007	1.163 **	1.364 ***
	(0.093)	(0.076)	(0.129)
Covariates adjusted	Yes	Yes	Yes
Dependent variable mean	0.165	0.349	0.089
R2	0.043	0.056	0.056
Obs	2503	3048	3048
Panel B:			
Adequate health knowledge	1.380 ***	1.131	0.922
	(0.158)	(0.093)	(0.127)
Doctors’ advice to quit	1.901 ***	2.067 ***	1.839 ***
	(0.222)	(0.171)	(0.258)
Self-reported good health (=1)	0.706 ***	0.768 ***	0.814
	(0.085)	(0.067)	(0.115)
Has hypertension (=1)	1.053	1.063	1.210
	(0.158)	(0.114)	(0.207)
Has heart diseases (=1)	0.494	0.972	0.517
	(0.271)	(0.329)	(0.323)
Has cerebrovascular … (=1)	0.873	2.150 **	3.768 ***
	(0.502)	(0.725)	(1.420)
Has cancer (=1)	1.000	4.126 **	6.478 ***
	(.)	(2.514)	(3.678)
Has other diseases (=1)	1.409	0.991	0.882
	(0.386)	(0.217)	(0.306)
Covariates adjusted	Yes	Yes	Yes
Dependent variable mean	0.165	0.349	0.089
R-squared	0.045	0.058	0.063
Obs	2496	3048	3048

Notes: (1) dependent variable in column 1 =1 if intend to quit in the next 12 months; dependent variable in column 2 =1 if attempted to quit in the past 12 months (former smokers included); dependent variable in column 3 =1 if quitted smoking in the past 12 months (former smokers included). (2) Covariates include survey year, gender, age, household size, annual household income per capita (log), education, job status and county dummies. (3) Exponentiated coefficients (odds ratios) are reported; standard errors in parentheses. ** *p* < 0.05, *** *p* < 0.01.

**Table A3:** Secondhand smoke and noticing smoking in public areas.

Variables	Rural		Urban	
	2018 (%)	2019 (%)	2018 (%)	2019 (%)
Adults who experienced				
… secondhand smoke	64.03	60.82	57.72	56.71
	(2811)	(2422)	(3756)	(3918)
… secondhand smoke daily	36.21	27.17	24.09	21.62
	(2811)	(2422)	(3756)	(3918)
Adults who noticed smoking				
… at workplaces	51.73	49.12	44.54	44.52
	(2107)	(1812)	(3116)	(3237)
… in hospitals	17.12	16.27	12.55	10.86
	(1858)	(1629)	(2638)	(2872)
… in government buildings	21.47	18.95	15.99	14.57
	(1085)	(955)	(1832)	(1874)
… in schools	9.54	12.95	7.47	10.34
	(1216)	(896)	(1969)	(1934)
… public transportations	/	14.00	/	14.97
	(0)	(1614)	(0)	(2999)

Source: NHLS survey in Ningbo, 2018, 2019. Note: numbers in parentheses indicate the number of observations in each entry.

**Table A4:** Logistic regression on varying cessation behaviours: full results.

Variables	(1)	(2)	(3)
	Intend to Quit 1y	Attempted 1y	Quitted 1y
	OR	OR	OR
Adequate health knowledge	1.392 ***	1.118	0.905
	(0.159)	(0.092)	(0.124)
Doctors’ advice to quit	1.876 ***	2.094 ***	1.903 ***
	(0.218)	(0.172)	(0.264)
Self-reported good health	0.713 ***	0.737 ***	0.748 **
	(0.083)	(0.062)	(0.102)
2019 vs. 2018	1.091	1.049	1.092
	(0.121)	(0.083)	(0.143)
Urban	0.863	0.940	1.099
	(0.108)	(0.085)	(0.160)
Male	0.563 *	0.602 **	0.306 ***
	(0.191)	(0.143)	(0.086)
Age/10	0.866 **	0.873 ***	0.852 **
	(0.051)	(0.038)	(0.059)
Household size	0.980	1.022	1.012
	(0.046)	(0.034)	(0.054)
Household annual income pc (log)	1.019	0.948 **	0.931 **
	(0.036)	(0.024)	(0.031)
Education level: illiterate	ref.	ref.	ref.
Elementary/middle	1.738*	1.059	0.637 *
	(0.556)	(0.200)	(0.171)
High school	1.476	0.997	0.437 **
	(0.518)	(0.216)	(0.142)
College or above	1.837	1.196	0.455 **
	(0.690)	(0.287)	(0.168)
Job status: public sectors	ref.	ref.	ref.
Farmers	0.654 *	0.642 **	0.723
	(0.149)	(0.111)	(0.200)
Manual labourers	0.581 **	0.785	0.785
	(0.131)	(0.132)	(0.215)
Other	0.750	0.786	0.972
	(0.150)	(0.122)	(0.243)
Covariates adjusted	Yes	Yes	Yes
Dependent variable mean	0.165	0.349	0.089
R-squared	0.043	0.055	0.051
N	2506	3052	3052

Notes: (1) dependent variable in column 1 =1 if intend to quit in the next 12 months; dependent variable in column 2 =1 if attempted to quit in the past 12 months (former smokers included); dependent variable in column 3 =1 if quitted smoking in the past 12 months (former smokers included). (2) Exponentiated coefficients (odds ratios) are reported; standard errors in parentheses. * *p* < 0.10, ** *p* < 0.05, *** *p* < 0.01.
